# Reply to: A Bisphosphonate With a Low Hydroxyapatite Binding Affinity Prevents Bone Loss in Mice After Ovariectomy and Reverses Rapidly With Treatment Cessation

**DOI:** 10.1002/jbm4.10492

**Published:** 2021-05-06

**Authors:** Abigail A Coffman, Jelena Basta‐Pljakic, Rosa M Guerra, Frank H Ebetino, Mark W Lundy, Robert J Majeska, Mitchell B Schaffler

**Affiliations:** ^1^ Biomedical Engineering City College of New York New York NY USA; ^2^ Pasadena CA USA; ^3^ Department of Biomedical Engineering The City College of New York New York NY USA

## Peer Review

The peer review history for this article is available at https://publons.com/publon/10.1002/jbm4.10492.

**To The Editor**

We thank Dr Pazianas for his comments emphasizing certain points raised in the discussion and conclusion of our article.^1^ There, we had endeavored to point out that this was a proof‐of‐concept study testing the utility of a rapidly reversible bisphosphonate (NE‐58025) in preventing bone loss. The only specific subsequent use for NE‐58025 that we endorsed at this very early stage of research was to explore the biomechanical consequences of a drug holiday. This obviously points to a preclinical research study in which such bone biomechanical studies can be performed. We further noted that short‐acting bisphosphonates that might require daily treatment could be problematic from a clinical perspective. We regret any confusion that might have resulted from our discussion.

The letter also offers several speculations about negative “off‐target” (i.e., outside of bone) effects caused by high circulating levels of low‐affinity bisphosphonate compounds, including the potential to develop osteonecrosis of the jaw. Off‐target effects are a concern with almost all drugs, and the specific points noted should be investigated in future studies designed to focus on these issues.

Finally, we note a potentially confusing typographical error. The letter refers to both NE‐58025 and NE‐58024; only one compound (NE‐58025) was studied in our research.

## Author Contributions

**Abigail Coffman:** Writing‐review & editing. **Jelena Basta‐Pljakic:** Writing‐review & editing. **Rosa Guerra:** Writing‐review & editing. **Frank Ebetino:** Writing‐review & editing. **Mark Lundy:** Writing‐review & editing. **Robert Majeska:** Writing‐review & editing. **Mitchell Schaffler:** Writing‐review & editing.
